# Inflammation and Lymphatic Function

**DOI:** 10.3389/fimmu.2019.00308

**Published:** 2019-02-26

**Authors:** Simon Schwager, Michael Detmar

**Affiliations:** Institute of Pharmaceutical Sciences, Swiss Federal Institute of Technology, ETH Zurich, Zurich, Switzerland

**Keywords:** lymphatic vessels, lymphangiogenesis, inflammation, inflammatory bowel disease, arthritis, psoriasis, skin, inflammatory mediators

## Abstract

The lymphatic vasculature plays a crucial role in regulating the inflammatory response by influencing drainage of extravasated fluid, inflammatory mediators, and leukocytes. Lymphatic vessels undergo pronounced enlargement in inflamed tissue and display increased leakiness, indicating reduced functionality. Interfering with lymphatic expansion by blocking the vascular endothelial growth factor C (VEGF-C)/vascular endothelial growth factor receptor 3 (VEGFR-3) signaling axis exacerbates inflammation in a variety of disease models, including inflammatory bowel disease (IBD), rheumatoid arthritis and skin inflammation. In contrast, stimulation of the lymphatic vasculature, e.g., by transgenic or viral overexpression as well as local injections of VEGF-C, has been shown to reduce inflammation severity in models of rheumatoid arthritis, skin inflammation, and IBD. Strikingly, the induced expansion of the lymphatic vasculature improves lymphatic function as assessed by the drainage of dyes, fluorescent tracers or inflammatory cells and labeled antigens. The drainage performance of lymphatic vessels is influenced by vascular permeability and pumping activity, which are influenced by VEGF-C/VEGFR-3 signaling as well as several inflammatory mediators, including TNF-α, IL-1β, and nitric oxide. Considering the beneficial effects of lymphatic activation in inflammation, administration of pro-lymphangiogenic factors like VEGF-C, preferably in a targeted, inflammation site-specific fashion, represents a promising therapeutic approach in the setting of inflammatory pathologies.

## Introduction

Inflammation is a defensive reaction of the organism against pathogens or irritants. It is characterized by the five cardinal symptoms of rubor (redness), calor (increased heat), tumor (swelling), dolor (pain), and functio laesa (impaired function), which are mostly mediated by the expansion and activation of blood vessels. Inflammation is commonly associated with the formation of new blood (angiogenesis) and lymphatic (lymphangiogenesis) vessels from the pre-existing vascular networks. Interestingly, while the activation of the blood vasculature has been reported to aggravate inflammation severity in a variety of disease models (1–3), lymphatic vessels generally appear to exert beneficial effects, possibly by improving the clearance of extravasated fluid, thus reducing edema formation and levels of pro-inflammatory mediators as well as numbers of immune cells.

This review provides an overview of studies investigating the role of lymphatic expansion and function in common inflammatory diseases such as skin inflammation, inflammatory bowel disease (IBD) and rheumatoid arthritis (RA). In addition, the known effects of inflammatory mediators on the lymphatic vasculature and commonly used mouse models are described.

The lymphatic vasculature is a hierarchically structured, one-way circuit composed of initial capillaries, which lack a continuous basement membrane and smooth muscle cell coverage, draining into larger, smooth muscle cell-covered collectors and ultimately lymph nodes. In the setting of inflammation, the lymphatic system is critically important, as it is needed to ensure tissue fluid homeostasis by draining the larger amounts of extravasated fluid originating from increasingly leaky, inflammatory blood vessels. Indeed, an increased interstitial fluid pressure has been found to lead to the dilation of initial lymphatic vessels, thus facilitating the entry of fluid and inflammatory cells into the lymphatic vasculature and thereby removal from the inflamed tissue (4). In addition, lymphatic vessels are crucial for immune surveillance, as they serve as main transport routes for cells and inflammatory mediators to lymph nodes, where immune responses are mounted.

The most-thoroughly characterized signaling axis involved in lymphatic expansion and development consists of the vascular endothelial growth factor receptor 3 (VEGFR-3) and its ligands VEGF-C and VEGF-D. VEGFR-3 is part of the receptor tyrosine kinase family and is expressed widely in vascular endothelial cells during embryonic development, but becomes strongly restricted to lymphatic endothelial cells (LECs) in the adult organism under physiological conditions (5).

VEGF-C is the main ligand of VEGFR-3 and induces proliferation and migration of endothelial cells (6, 7). It undergoes extensive post-translational proteolytic processing, which also regulates the molecule's binding properties. Fully processed VEGF-C binds VEGFR-3 and, albeit with a lower affinity, VEGFR-2 (8). A mutated form of VEGF-C in which the cysteine 156 is replaced with a serine (VEGF-C156Ser) selectively binds VEGFR-3 (9).

VEGF-D has been reported to induce proliferation of endothelial cells (10). In mice, VEGF-D exclusively binds VEGFR-3, while fully processed human VEGF-D may also bind VEGFR-2 (11).

In order to study the role of lymphatic vessels in different pathologies, various mouse lines with a modified VEGFR-3 signaling axis have been generated. In K14-VEGF-C mice, VEGF-C is overexpressed under the control of the keratin-14 promoter, resulting in elevated levels of the growth factor in the skin and an enlarged dermal lymphatic vascular network (12). A similar lymphatic hyperplasia has been observed in mice transgenic for VEGF-D (K14-VEGF-D) (13). In contrast, mice overexpressing a soluble form of VEGFR-3 in the skin (K14-VEGFR-3-Ig mice) lack dermal lymphatic vessels and develop edema in the feet and skin (14).

Apart from promoting or inhibiting lymphatic vascular expansion, the clearance capacity of lymphatic vessels is subject to regulation by various signals. Drainage performance is influenced by vascular permeability and pumping activity of lymphatic vessels. Mediators inducing increased lymphatic vessel permeability include TNF-α, IL-1β, histamine, and the VEGF-C/VEGFR-3 axis (15–17). Lymphatic contractions and thereby pumping are negatively regulated by various inflammatory mediators, including prostaglandins, histamine, and nitric oxide (NO), while VEGF-C has enhancing effects (18–21). NO regulates lymphatic vessel function via its effects on lymphatic smooth muscle cells leading to vasodilation. It is produced constitutively by the endothelial nitric oxide synthase (eNOS) under physiological conditions. In inflammation, however, its levels are elevated due to the higher expression of inducible nitric oxide synthase (iNOS) on immune cells and inflamed endothelium, which has been linked to reduced lymphatic contraction frequency (22).

## The Lymphatic Vasculature in Inflammatory Diseases

### Skin Inflammation

A wide range of skin pathologies including psoriasis, atopic dermatitis, rosacea, and UV damage are characterized by pronounced and often prolonged inflammation. The lymphatic vasculature is often aberrant in inflamed skin; in human psoriatic plaques for example, lymphatic vessels are dilated and tortuous (23–25). Nevertheless, lymphatic dysregulation in the human disease has attracted comparatively little attention.

Multiple mouse models have been established to facilitate the study of these diseases in general and the role of the lymphatic vasculature in particular. A common model are K14-VEGF-A transgenic mice which overexpress VEGF-A under the control of the keratin-14 promoter, resulting in chronically elevated levels of said growth factor in the skin and a concomitant expanded, leaky blood vasculature. Homozygous mice spontaneously develop a chronic skin inflammation at the age of 6 months (26). In hemizygous mice, a contact sensitizer (e.g., oxazolone) can be used to trigger a contact hypersensitivity reaction (CHS), leading to a similar chronic inflammatory skin disease (24).

In wild-type mice, skin inflammation may be elicited by inducing CHS, exposure to UVB radiation, injection of bacterial antigens like LPS or application of pro-inflammatory agents such as tetradecanoylphorbolacetate (TPA) or imiquimod (27).

Using these models, skin inflammation has been extensively studied in mice and the lymphatic vasculature has been demonstrated to be functionally impeded in UVB-irradiated, chronically inflamed ear skin. Evans blue injected into the inflamed skin stained strongly dilated lymphatic vessels that were extremely leaky, indicating reduced drainage capacity (1).

#### Stimulation of Lymphatic Vessels in Skin Inflammation

Activating the lymphatic vasculature in the setting of skin inflammation has been associated with reduced disease severity (summarized in Table 1). In K14-VEGF-A mice that had been crossed with K14-VEGF-C mice and were undergoing chronic CHS of the ear skin, the lymphatic vasculature was expanded and the inflammation, as assessed by edema formation, inflammatory cell infiltrate, and altered epidermal proliferation or differentiation, was significantly reduced compared to control inflamed K14-VEGF-A mice. Strikingly, the vascular expansion was accompanied by an improved lymphatic clearance function. Local injections of VEGF-C156Ser had similar disease-alleviating effects, indicating that VEGFR-3- rather than VEGFR-2-mediated signaling is mainly responsible for the observed anti-inflammatory effects (28). In agreement with this observation, local injections of VEGF-C156Ser also triggered a strong lymphangiogenic response and reduced inflammatory ear swelling and CD11b-positive immune cell infiltration in UVB-irradiated ear skin inflammation (32).

**Table 1:** Effects of lymphatic vessel stimulation in inflammatory diseases.

**Animal model**	**Inflammatory stimulus**	**Method of lymphatic vasculature activation**	**Effects**	**References**
**SKIN INFLAMMATION**				
K14-VEGF-A mice	Oxazolone	Transgenic VEGF-C delivery (crossed with K14-VEGF-C mice)	Reduced inflammatory edema and cell infiltration Expanded skin lymphatic vasculature Normalization of skin blood vasculature, epidermal differentiation and proliferation Improved lymphatic drainage function	(28)
		Local injection of recombinant VEGF-C156Ser	Reduced inflammatory edema Expanded skin lymphatic vasculature Normalization of skin blood vasculature Reduced inflammatory cell infiltration	
K14-VEGF-C mice	Injections of LPS or LTA and MDP	Transgenic VEGF-C delivery	Expanded lymphatic skin and LN vasculature Increased inflammatory cell migration to LNs Reduced inflammatory edema and erythema Faster antigen clearance	(29)
	TPA		Increased clearance of lymphatic-specific tracer	(30)
	UVB irradiation		Reduced inflammatory edema and epidermal thickening Expanded lymphatic vasculature Improved lymphatic drainage function	(31)
	Oxazolone		Reduced inflammatory edema and epidermal thickening Expanded lymphatic vasculature Lower levels of IL-1β and VEGF-A	
K14-VEGF-D mice	UVB irradiation	Transgenic VEGF-D delivery	Reduced inflammatory edema and epidermal thickening Expanded lymphatic vasculature Improved lymphatic drainage function	(31)
	Oxazolone		Reduced inflammatory edema and epidermal thickening Expanded lymphatic vasculature	
Wildtype mice	UVB irradiation	Local injection of recombinant VEGF-C156Ser	Reduced inflammatory edema and cell infiltration Expanded lymphatic vasculature	(32)
**INFLAMMATORY BOWEL DISEASE**				
Wildtype mice	DSS	Adenoviral delivery of VEGF-C	Reduced colitis severity and inflammatory cell infiltration Increased lymphatic vessel density and proliferation Improved lymphatic drainage function Increased inflammatory cell migration to LNs	(33)
IL-10 knockout mice	Lack of anti-inflammatory IL-10			
**RHEUMATOID ARTHRITIS**				
TNF-α transgenic mice	TNF-α overexpression	Adeno-associated viral delivery of VEGF-C	Expanded lymphatic vasculature Reduced synovial volume, bone and cartilage erosion and osteoclast numbers Improved joint movement and lymphatic clearance function	(34)
		iNOS inhibition	Improved lymphatic clearance function Restored lymphatic contractions	(35)

*DSS, dextran sulfate sodium; LN, lymph node; LPS, lipopolysaccharide; LTA, lipoteichoic acid; LV, lymphatic vessel; MDP, muramyl dipeptide; TPA, tetradecanoylphorbolacetate*.

These findings are in line with a different study investigating the role of macrophages and lymphatic vessels in cutaneous inflammation. K14-VEGF-C mice that were subjected to lipopolysaccharide (LPS)- or lipoteichoic acid (LTA)/muramyl dipeptide (MDP)-induced skin inflammation presented with an expanded dermal and lymph node lymphatic vasculature. In addition, inflammatory tissue swelling and skin reddening were reduced. While no difference in FITC-dextran clearance was found, inflammatory cell migration to the draining lymph nodes and the drainage of fluorescently labeled antigen was significantly accelerated in K14-VEGF-C mice. These effects appeared to be dependent on macrophages, as clodronate-mediated depletion of these cells reduced lymphangiogenesis and delayed inflammation resolution (29). An enhanced lymphatic drainage function due to lymphatic stimulation has also been reported in other studies, e.g., after repeated application of TPA to the back skin of K14-VEGF-C transgenic mice, in which a lymphatic-specific, near-infrared tracer was cleared more rapidly than in wild-type mice (30). Similarly, in a study of acute skin inflammation, both K14-VEGF-C and, to a lesser extent, K14-VEGF-D transgenic mice had improved clearance of Evans blue out of UVB-irradiated ear skin (31). Moreover, these mice also had less inflammatory edema and reduced epidermal thickening in oxazolone- and UVB-induced skin inflammation. The reduction in inflammation was generally more pronounced in VEGF-C transgenic mice than in VEGF-D transgenic animals, indicating stronger anti-inflammatory effects of VEGF-C (31).

#### Inhibition of Lymphatic Vessels in Skin Inflammation

In contrast to stimulation of the lymphatic vasculature, inhibiting lymphatic vessels has been shown to aggravate skin inflammation in several studies (summarized in Table 2). Antibody-mediated blocking of VEGFR-3 strikingly reduced the number of lymphatic vessels in the inflamed ear skin of K14-VEGF-A mice during a CHS reaction. At the same time, tissue swelling, epidermal thickening, keratinocyte proliferation and the numbers of CD8- and CD11b-positive cells were significantly increased, indicating a more severe inflammatory phenotype. Interestingly, blocking VEGFR-2 alone or in combination with VEGFR-3 alleviated inflammation, indicating that VEGFR-2-mediated inhibition of blood vessels is beneficial in skin inflammation and outweighs the detrimental effects of VEGFR-3 inhibition (28). Similarly, adenoviral overexpression of a soluble VEGFR-3 strongly reduced lymphangiogenesis in mice undergoing LPS- or LTA/MDP-induced skin inflammation, resulting in delayed inflammation-resolution, slower clearance of FITC-dextran as well as FITC-labeled LPS, and reduced migration of inflammatory cells from the skin to the draining lymph nodes (29). Systemic, antibody-mediated inhibition of VEGFR-3 also led to increased edema formation and CD11b-positive cell numbers in UVB-irradiated ear skin (36).

**Table 2:** Effects of lymphatic vessel inhibition in inflammatory diseases.

**Animal model**	**Inflammatory stimulus**	**Method of lymphatic vasculature inhibition**	**Effects**	**References**
**SKIN INFLAMMATION**				
K14-VEGF-A mice	Oxazolone	Blocking antibody to VEGFR-3	Reduced lymphatic vasculature Increased inflammatory edema and epidermal thickening	(28)
Wild-type mice	Injections of LPS or LTA and MDP	Adenoviral VEGFR-3 overexpression	Delayed inflammation resolution Reduced lymphatic drainage and inflammatory cell migration	(29)
	UVB irradiation	Blocking antibody to VEGFR-3	Increased inflammatory edema and inflammatory cell invasion	(36)
**INFLAMMATORY BOWEL DISEASE**				
Wildtype mice	DSS	Blocking antibody to VEGFR-3	Increased colitis severity Reduced lymphatic vessel density, LV proliferation, lymphatic drainage function and cell migration to LN	(33)
IL-10 knockout mice	Lack of anti-inflammatory IL-10	Blocking antibody to VEGFR-3	Increased colitis severity Reduced lymphatic vessel density, LV proliferation, lymphatic drainage function and cell migration to LN	(33)
			Increased colitis severity and edema Enlarged lymphatic vessels	(37)
**RHEUMATOID ARTHRITIS**				
TNF-α transgenic mice	TNF-α overexpression	Blocking antibody to VEGFR-3	Reduced lymphatic vessel numbers and lymphatic drainage Smaller draining LNs Increased joint inflammation	(38)

*DSS, dextran sulfate sodium; LN, lymph node; LPS, lipopolysaccharide; LTA, lipoteichoic acid, LV, lymphatic vessel; MDP, muramyl dipeptide*.

### Inflammatory Bowel Disease

The term inflammatory bowel disease (IBD) comprises Crohn's disease (CD) and ulcerative colitis (UC), which are characterized by a chronic inflammation of the digestive tract. While UC generally affects the colon and presents with superficial ulcerations of the mucosa and submucosa, CD may occur at any location in the gastrointestinal tract and often causes transmural inflammation. As in the case of skin inflammation, research has long been focused on changes in the blood vasculature and VEGF-A has been suggested as an important mediator of IBD (39, 40).

In human patients suffering from IBD, lymphangiogenesis, lymphatic vessel obstruction, dilation, and submucosal edema are commonly observed (41–44) and abnormalities in the lymphatic vasculature had already been recognized during the original characterization of CD (45). In addition to morphological alterations, the functionality of IBD-associated lymphatic vessels is reduced. A study in patients with CD employed injections of the lymphatic-vessel-staining Patent Blue V dye in the inflamed colon and demonstrated morphological aberrations and functional impairment of the lymphatic vasculature, which could be correlated with disease severity. Strikingly, following surgical intervention and inflammation regression, lymphatic vessel appearance reverted back to normal, indicating that lymphatic vessel function may be involved in IBD pathogenesis in humans (46). In line with this, a lower density of lymphatic vessels could be linked to an increased risk of CD recurrence (47).

A multitude of studies have been performed in mouse models of IBD, the two most-commonly used being IL-10 knockout mice and dextran sulfate sodium (DSS)-induced colitis. IL-10-deficient mice spontaneously develop colitis at the age of 10–12 weeks, most likely due to the lacking anti-inflammatory and immunosuppressive activity of IL-10 (48, 49).

DSS-induced colitis relies on administration of the detergent DSS in drinking water, which damages the intestinal epithelium, most strongly in the distal colon, and compromises its barrier function, making the underlying tissue accessible to bacteria and associated substances. In order to model acute inflammation, mice are commonly given DSS for a certain amount of time (e.g., a week), for chronic inflammation, mice receive multiple cycles of DSS and intermittent regular drinking water (50, 51).

#### Stimulation of Lymphatic Vessels in Inflammatory Bowel Disease

Akin to skin inflammation, inducing the lymphatic vasculature is generally correlated with a reduction in inflammation severity (summarized in Table 1).

In IL-10 knockout mice as well as in animals undergoing DSS-induced colitis, adenoviral delivery of VEGF-C significantly increased lymphatic vessel density and was associated with a reduction in bodyweight loss and disease severity as assessed by stool consistency and presence or absence of fecal blood. Moreover, histological analyses revealed decreased submucosal tissue edema and inflammatory cell infiltration, while the proliferation of LECs was greatly increased. Quantification of Evans blue clearance out of inflamed distal colon tissue revealed an enhanced lymphatic drainage function, which was also reflected in an improved clearance of fluorescently labeled antigen-coated beads and an augmented inflammatory cell migration from the inflamed tissue to the draining lymph nodes. Similar to the observations in skin inflammation, depletion of macrophages by clodronate largely abolished the protective effects of VEGF-C (33). It has been suggested that VEGF-C may influence the cytokine balance in the inflamed colon. Indeed, *in vitro* experiments have shown VEGF-C to induce the upregulation of IL-10 by bone marrow-derived macrophages (33). In line with this, increased levels of IL-10 in combination with a reduction of IL-9, which is associated with intestinal barrier disruption, have been reported upon treatment with adenovirally delivered VEGF-C in mice undergoing DSS-induced colitis (52, 53).

#### Inhibition of Lymphatic Vessels in Inflammatory Bowel Disease

Blocking VEGFR-3 resulted in a worsened colitis in IL-10 knockout mice as well as DSS-treated animals in terms of the histological score (summarized in Table 2). Animals of both models presented with strongly reduced lymphatic vessel density and LEC proliferation upon VEGFR-3 inhibition. At the same time, lymphatic clearance of Evans blue and bacterial antigen as well as inflammatory cell mobilization to the draining lymph nodes were significantly reduced (33).

In a different, independent study, IL-10 knockout mice were treated with a blocking antibody to VEGFR-3. This resulted in enlarged and tortuous lymphatic vessels in the colon, increased submucosal edema and a higher leukocyte infiltration in the inflamed tissue as well as a higher disease severity score (37).

### Rheumatoid Arthritis

Rheumatoid arthritis (RA) is a chronic inflammatory disease affecting the joints and characterized by episodic flares (54). In its chronic stage, RA is commonly associated with lymphadenopathy and a decrease in lymphatic drainage function, as shown for example by tracking the drainage of intradermally-injected, radioactively labeled albumin from the forearm (55). Lymphangiogenesis is also commonly observed in the joints of human RA patients and has been reproduced in mouse models of the disease (56, 57).

Commonly used mouse models of rheumatoid arthritis include TNF-α transgenic mice and K/B × N mice. The former overexpress human TNF-α and spontaneously develop chronic progressive joint inflammation at the age of ~4 weeks (58). K/B × N mice model the autoimmunity aspect of RA and are based on a mouse line transgenic for a T cell receptor specific for bovine ribonuclease. After breeding onto the NOD background, accidental recognition of a NOD-derived antigen triggers the onset of joint inflammation at 4 weeks after birth (59).

Lymphatic function has mainly been studied in these animals and a two-phase model has been proposed [reviewed in (60)]. In an initial “expansion” phase during joint inflammation, lymphangiogenesis and popliteal lymph node expansion with or without increased lymphatic vessel contractions limit the inflammatory response (57, 61, 62). During the following “collapse” phase, popliteal lymph nodes shrink and lymphatic vessel contractions as well as lymphatic drainage function decrease significantly. At the same time, the joint inflammation increases in severity (61, 63–65). Blocking TNF-α signaling resulted in increased lymphatic contractions and reduced joint inflammation (66).

Similar changes in lymph node characteristics have also been reported in human patients, where lymph node hypertrophy could be observed in the vast majority of patients suffering from active RA, while healthy individuals and patients in remission showed no lymph node alterations (67).

#### Stimulation of Lymphatic Vessels in Rheumatoid Arthritis

Stimulating the lymphatic vasculature has been associated with reduced disease severity in animal models of RA (summarized in Table 1). Adeno-associated viral (AAV) delivery of VEGF-C in the inflamed ankle joints of 6-week-old TNF-α transgenic mice partially reversed the inflammation-associated increase in synovial volume and significantly improved leg mobility. Histological analyses revealed that mice treated with VEGF-C had less cartilage and bone destruction than animals injected with a control vector. In chronic arthritis (mice at 5 months of age), lymphatic drainage of indocyanine green (ICG) out of the footpad was strongly decreased in TNF-α transgenic compared to wild-type mice. AAV-mediated delivery of VEGF-C significantly improved the clearance of ICG out of the paws and increased the number of lymphatic vessels in the pannus of the inflamed joint (34).

In an alternative approach, based on the observation that increased levels of NO in inflammation reduce lymphatic pumping, lymphatic vessel function was studied using inhibition of NOS. Local application of L-N6-(1-iminoethyl)lysine 5-tetrazole-amide (L-NIL), a moderately selective inhibitor of iNOS (68), in TNF-α transgenic mice with collapsed lymph nodes restored lymphatic contractions and strongly improved ICG transport from the footpad to popliteal lymph nodes, while Nω-nitro-l-arginine methyl ester (L-NAME), an unspecific inhibitor of both eNOS and iNOS was not associated with beneficial effects (35). Although the impact on disease severity in these mice was not assessed in the study, it provides evidence that selective inhibition of iNOS might offer an alternative and clinically relevant approach for RA therapy.

#### Inhibition on Lymphatic Vessels in Rheumatoid Arthritis

Inhibiting the lymphatic vasculature led to worsened inflammation in mouse models of arthritis (summarized in Table 2). Injecting TNF-α transgenic mice that had developed joint inflammation with a VEGFR-3-blocking antibody for 2 months significantly reduced the number of lymphatic capillaries in the draining popliteal lymph nodes and inflamed ankles. Blocking VEGFR-3 also aggravated inflammation of the knee and ankle joints, as the increase in synovial volume over time as well as its absolute size were elevated in these animals compared to IgG-treated controls. Similarly, histological analyses of hematoxylin-eosin-stained sections revealed exacerbated inflammation after VEGFR-3 inhibition. Akin to the effects observed in chronic skin inflammation, blocking VEGFR-2 was associated with a reduced inflammatory reaction, as assessed by synovial volume and histological scoring. Lymphatic drainage function, as assessed by tracking the ICG signal in paws and draining popliteal lymph nodes following injection into the footpad, was dramatically reduced upon blocking VEGFR-3 (38).

### The Effect of Inflammatory Mediators on the Lymphatic Vasculature

Inflammatory lymphangiogenesis is mostly mediated by VEGF-A and VEGF-C which are produced by keratinocytes and stromal cells like fibroblasts as well as immune cells, most importantly macrophages (69–71). Indeed, several inflammatory mediators have been found to induce VEGF-C transcription (72–74).

Macrophages are of critical importance, as demonstrated in a model of IBD and LPS-induced skin inflammation, where depletion of macrophages aggravated the inflammation (29, 33). While VEGFs are important for inflammation-induced lymphangiogenesis, there are many additional factors at play. IL-17, a crucial cytokine in the pathogenesis of psoriasis for example, has been shown to induce lymphangiogenesis *in vitro* and in cornea micropocket assays (75), and IL-8 promoted lymphangiogenesis in cell culture experiments and in an animal model of lymphedema (76). Similarly, inhibition of TGF-β, which mediates anti-inflammatory effects, supported lymphangiogenesis in a mouse model of peritonitis and in lymphedema (77, 78). In line with this, cytokines characteristic for T_H_2 cells like IL-4 and IL-13, which are often linked to inflammation resolution, inhibited lymphangiogenesis (79). Interestingly, several inflammatory mediators have anti-lymphangiogenic activity. Interferon-γ (IFN-γ), which is produced by activated T cells, decreased lymphatic vessel formation of both human and murine lymphatic endothelial cells *in vitro* as well as in mouse lymph nodes (15, 80). Likewise, TNF-α inhibited capillary formation and proliferation of mouse LECs, while IL-1β had no consistent effects on proliferation, but reduced barrier function of LECs (15). Indeed, inflammatory mediators not only influence lymphangiogenesis, but also impact lymphatic function more directly. Prostaglandins, IL-1β, IL-6, and TNF-α reduced lymphatic pumping frequency (81, 82). Similarly, inflammatory mediators affect lymphatic vessel permeability, as demonstrated *in vitro* by assessing the effect of a wide array of inflammatory mediators on rat lymphatic endothelial cell monolayers, where IL-6, TNF-α, and IFN-γ strikingly increased the permeability, probably by reducing vascular endothelial (VE)-cadherin expression (83). Few studies have addressed lymphatic vessel permeability *in vivo*, but results of those that have showed impaired barrier function as well as pronounced leakiness and reported VEGF-A as important mediator of these effects, possibly by signaling via VEGFR-2 (1, 84).

It is important to consider that cytokines and growth factors often have pleiotropic effects, making it challenging to distinguish between direct and indirect mechanisms. IL-17 for example has been reported to induce VEGF-D expression, thereby triggering lymphangiogenesis indirectly (75). The wide array of signaling molecules involved in inflammation as well as their different and often pleiotropic effects on the lymphatic vasculature result in a highly complex network of signals which is still incompletely understood.

## Conclusions

**Figure 1:**
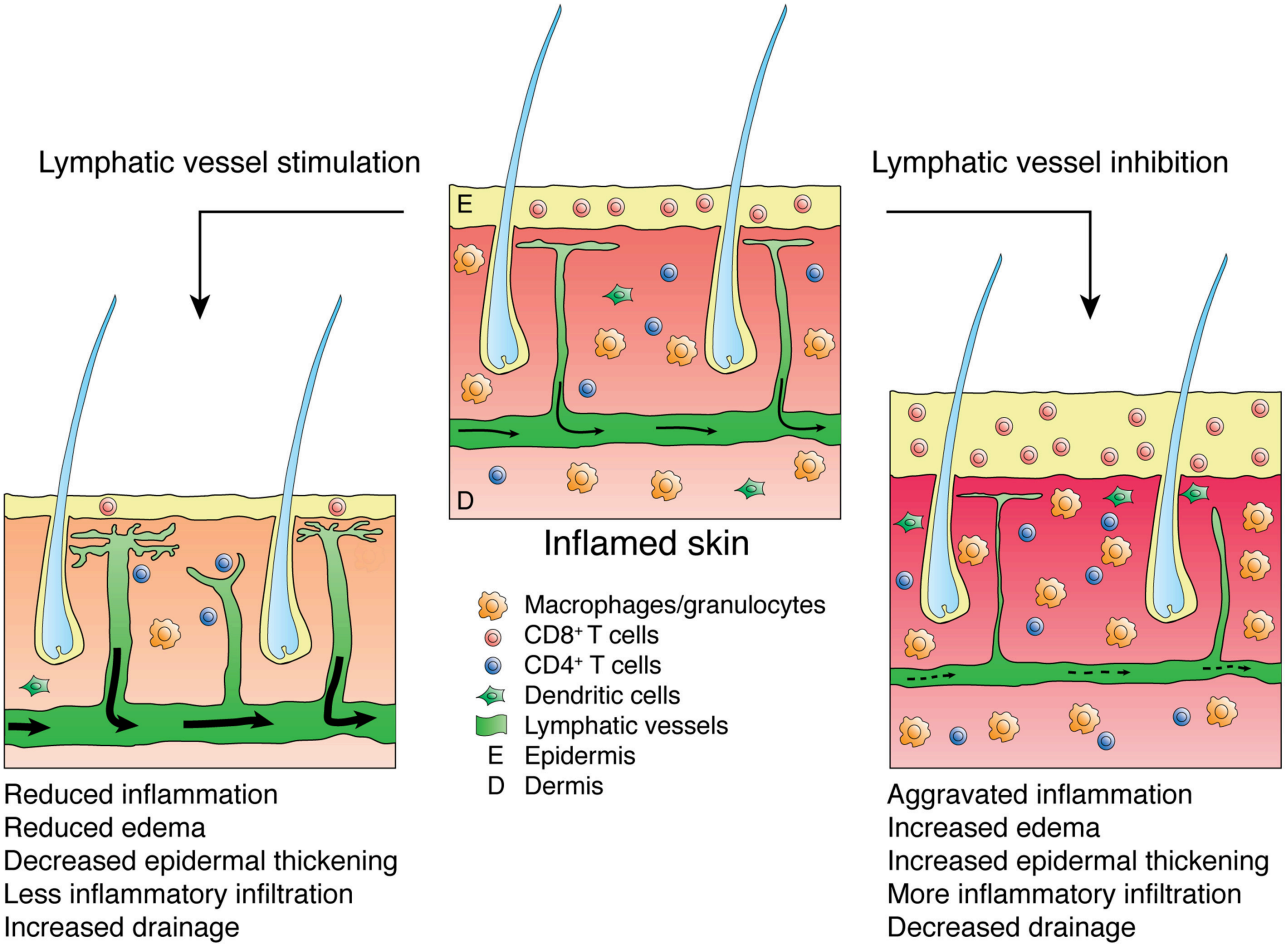
Effects of lymphatic vessel stimulation or inhibition on skin inflammation. Inflamed skin presents with epidermal thickening, edema and infiltration by inflammatory leukocytes (e.g., CD8-positive cells or macrophages and granulocytes). Stimulation of the lymphatic vasculature alleviates inflammation, reducing edema, epidermal thickening and inflammatory infiltration while improving lymphatic drainage, thus lowering the numbers of inflammatory cells in the inflamed skin. Inhibition of the lymphatic vasculature aggravates inflammation and reduces lymphatic clearance.

The lymphatic vasculature represents a crucial, although often under-appreciated, player in inflammation. Lymphatic vessels serve as the main transport route for inflammatory mediators, fluid, antigen and immune cells, thus playing a pivotal role in inflammation initiation and resolution. Indeed, it has been controversial whether expansion of the lymphatic endothelium contributes to inflammation by facilitating transport of leukocytes to lymph nodes and mounting of immune responses, or whether lymphatic vessels support inflammation resolution by draining inflammatory mediators and cells from the site of inflammation. However, in recent years, a number of studies detailed above have reported alleviated inflammation severity following activation and/or expansion of the lymphatic vasculature (depicted for skin inflammation in Figure 1), thus indicating that promoting the lymphatic vasculature supports inflammation resolution and may represent a valid therapeutic approach. It should be considered, however, that VEGF-C/VEGFR-3 signaling itself might also account for some of the anti-inflammatory effects observed in VEGF-C transgenic mice, as it has been shown to reduce the production of pro-inflammatory cytokines and protect mice from septic shock (85).

Interestingly, the lymphatic vasculature is also affected by established standard therapies used for the treatment of inflammatory diseases, e.g., in RA, where blocking TNF-α resulted in an increased lymphangiogenic response and increased lymphatic contractions in the inflamed tissue (66, 86). Other therapies aimed at blocking certain cytokines (e.g., IL-17 in psoriasis) may also exert parts of their anti-inflammatory effects by modulating the lymphatic vasculature. Curiously, some anti-inflammatory agents have been associated with anti-lymphangiogenic activity. Glucocorticoids reduced lymphangiogenesis in cornea inflammation and chronic airway inflammation mediated by *M. pulmonis* infection (87, 88). In addition, prostaglandin E2, whose biosynthesis is inhibited by cyclooxygenase (COX)-blocking non-steroidal anti-inflammatory drugs (NSAIDs), has been reported to induce VEGF-C expression and lymphangiogenesis in the setting of lung cancer (73). Coherently, inhibition of COX-2 reduced tumor-induced lymphangiogenesis (89). A possible explanation for these findings could be that potent therapeutic agents inhibit inflammation strongly enough to also reduce the concomitant inflammation-induced lymphangiogenesis. Moreover, while prostaglandin E2 interferes with lymphatic expansion, it has also been reported to inhibit lymphatic function (81). Therefore, glucocorticoids and NSAIDs may improve lymphatic clearance despite reducing lymphangiogenesis. However, further studies are needed to thoroughly investigate these possibilities.

It is important to consider that immunomodulatory properties of the lymphatic endothelium, which have received increasing attention over the last decade, may explain the observed anti-inflammatory effects of lymphatic vessel induction at least partially. A good example is the receptor D6, which is highly expressed by lymphatic endothelial cells and scavenges inflammatory cytokines. Mice deficient for D6 suffered from more severe skin inflammation and colitis compared to wild-type animals (90, 91), hence, lymphatic expansion may increase the levels of D6 and accordingly lower the levels of inflammatory mediators in the inflamed tissue, resulting in reduced disease severity. However, the immunomodulatory roles of the lymphatic vasculature are outside the scope of this review.

Although VEGF-C has been associated with anti-inflammatory effects in a variety of diseases as described above, its biological roles are highly complex and may be organ- and disease-dependent. In the setting of experimental obesity for example, transgenically overexpressed VEGF-C induced pro-inflammatory macrophage chemotaxis, increased weight gain and worsened metabolic parameters such as insulin resistance (92). In contrast, blockade of VEGF-C and VEGF-D by overexpression of a soluble form of VEGFR-3 reduced macrophage infiltration and improved insulin sensitivity in diet-induced obesity (93). Similarly, in tumor studies, VEGF-C has been reported to induce tumor lymphangiogenesis and stimulate the migration of macrophages (94), which may explain the observed increase in tumor metastasis in VEGF-C transgenic mice (95).

Applying VEGF-C in these diseases might be counter-productive and these findings therefore highlight the complexity of VEGF-C biology and emphasize the necessity of thoroughly evaluating possible beneficial and detrimental effects of VEGF-C in individual pathologies.

Considering all available data, the induction of lymphangiogenesis and activation of the lymphatic vasculature in the setting of inflammation appears to represent a potent therapeutic approach. It is therefore striking that this strategy has not been explored more thoroughly, let alone exploited clinically. A major obstacle has been the lack of clinically feasible delivery systems of lymphangiogenic factors. In a recent study, however, a targeted F8-VEGF-C fusion protein that specifically accumulates in the inflamed tissue was characterized and shown to reduce inflammation in two mouse models of skin inflammation, possibly filling this therapeutic gap (96).

## Author Contributions

SS designed and wrote the manuscript. MD designed and revised the manuscript.

### Conflict of Interest Statement

The authors declare that the research was conducted in the absence of any commercial or financial relationships that could be construed as a potential conflict of interest.
